# Data on the exon-intron organization of genes coding for B-cell receptor-like proteins

**DOI:** 10.1016/j.dib.2017.05.011

**Published:** 2017-05-06

**Authors:** Sergey Y. Morozov, Anna V. Pankratenko, Anastasia K. Atabekova, Andrey G. Solovyev

**Affiliations:** Department of Virology and A. N. Belozersky Institute of Physico-Chemical Biology, Lomonosov Moscow State University, 119992 Moscow, Russia

**Keywords:** B-cell receptor protein, Gene organization, Exons, Introns, Unikonts, Bikonts

## Abstract

B-cell receptor-associated protein (BAP) family plays important roles in the ER homeostasis and stress responses of eukaryotic cells [1]. We reported the analysis of plant BAP-like (PBL) genes and the encoded proteins of higher land plants [2]. The origin and functional divergence of these genes among all eukaryotes, however, are poorly studied, which impedes our understanding of the functional relationships and diversity among BAP-like proteins. One possible reason for the potential functional diversity may be the differences in the exon-intron structure of PBL genes. In this study, we first performed analysis of the exon-intron organization of these genes in the genome sequences of the Viridiplantae species in addition to previously published data on Angiosperms [2]. To further address the distribution of BAP-like genes in other eukaryotes, we extended our dataset to include the representative genes encoded by non-plant bikonts and unikonts [3].

**Specifications Table**

**Table:** 

Subject area	*Biology*
More specific subject area	*Phylogenomics*
Type of data	*Table*
How data was acquired	*Gene sequence organizations and protein parameters were derived using ExPASy software, NCBI, Dendrome and Phytozome databases (see below)*
Data format	*Analyzed*
Experimental factors	*Amino acid and nucleotide sequences were retrieved from NCBI, Dendrome and Phytozome databases (see below).*
Experimental features	*Gene and protein sequences were derived using NCBI, Dendrome and Phytozome databases (see below)*
Data source location	NCBI: http://www.ncbi.nlm.nih.gov/
	Phytozome v11: http://www.phytozome.net/
	ExPASy: http://www.expasy.org/tools/
	Dendrome: http://dendrome.ucdavis.edu/
Data accessibility	*With this article*

**Value of the data**

• The BAP-like genes are usually combined in small gene families and encoded by all well-studied eukaryotic taxons belonging to bikonts and unikonts.
• Data on BAP-like gene organizations enable researchers to compare how these genes evolved during progression of different eukaryotic branches resulted, particularly, in appearance of mammals and flowering plants.
• Data on BAP-like proteins and genes are intriguing to understand their unique features in different taxons.
• Data on gene organizations enable researchers to infer the possible ranges of time frames in the drastic divergence events (particularly, intron gain and loss) of BAP-like genes.

## 1 Data and experimental design

Annotation information was obtained for 29 representative genes in bikonts (Table 1) and 81 genes in unikonts (Table 2) [[1], [2], [3]]using Blast2GO program.

**Table 1:** Features of BAP-like proteins and genes in the selected Biconta species^a^.

**Species name**	**Features of proteins and genes**				
**NCBI accession number**	**Taxon**	**Protein length**	**ProteinpI**	**Number of introns**	**Length of introns**
*Amborella trichopoda* (**NW_006496807**)	**Magnoliophyta Amborellales**	**220 aa**	**9.18**	**1**	**19293 bp**
*Amborella trichopoda* (**NW_006499207**)	**Magnoliophyta Amborellales**	**220 aa**	**9.88**	**1**	**14781 bp**
*Pinus lambertiana* (**LMTP010039273**)	**Acrogymnospermae Pinales**	**228 aa**	**9.61**	**1**	**82221 bp**
*Pseudotsuga menziesii* (**LPNX010213588**)	**Acrogymnospermae Pinales**	**228 aa**	**9.59**	**1**	**71087 bp**
*Pinus taeda* (**APFE020507172**)	**Acrogymnospermae Pinales**	**230 aa**	**9.37**	**1**	**66410 bp**
*Selaginella moellendorffii* (**NW_003314330**)	**Lycopodiopsida Selaginellales**	**233 aa**	**9.46**	**1**	**68 bp**
*Physcomitrella patens* (**XP_001766710**)	**Bryopsida Funariales**	**257 aa**	**9.40**	**2**	**222 bp; 71 bp**
*Sphagnum fallax* (**Sphfalx0532s0001**^b^)	**Sphagnopsida Sphagnales**	**238 aa**	**9.58**	**2**	**996 bp; 329 bp**
*Sphagnum fallax* (**Sphfalx0012s0168**^b^)	**Sphagnopsida Sphagnales**	**238 aa**	**9.58**	**2**	**992 bp; 329 bp**
*Sphagnum fallax* (**Sphfalx0223s0006**^b^)	**Sphagnopsida Sphagnales**	**213 aa**	**9.90**	**2**	**1010 bp; 331 bp**
*Sphagnum fallax* (**Sphfalx0012s0164**^b^)	**Sphagnopsida Sphagnales**	**210 aa**	**9.28**	**2**	**1013 bp; 331 bp**
*Marchantia polymorpha* (**OAE31879**)	**Marchantiopsida Marchantiales**	**189 aa**	**5.91**	**2**	**140 bp 681 bp**
*Klebsormidium flaccidum* (**BANV01001480**)	**Klebsormidiophyceae Klebsormidiales**	**247 aa**	**8.67**	**2**	**487 bp 330 bp**
*Chlorella variabilis* (**XP_005842886**)	**Chlorophyta Chlorellales**	**291 aa**	**5.64**	**5**	**176 bp 191 bp 98 bp 189 bp 255 bp**
*Chlamydomonas reinhardtii* (**XP_005842886**)	**Chlorophyta Chlamydomonadales**	**259 aa**	**9.11**	**5**	**291 bp 264 bp 327 bp 352 bp 171 bp**
*Monoraphidium neglectum* (**XP_013903892**)	**Chlorophyta Sphaeropleales**	**232 aa**	**9.48**	**3**	**182 bp 190 bp 243 bp**
*Volvox carteri* (**XP_002952600**)	**Chlorophyta Chlamydomonadales**	**255 aa**	**9.30**	**4**	**166 bp 752 bp 2055 bp 434 bp**
*Galdieria sulphuraria* (**XP_002952600**)	**Rhodophyta Cyanidiales**	**254 aa**	**9.91**	**4**	**46 bp 51 bp 56 bp 53 bp**
*Phytophthora infestans* (**XP_002898022**)	**Oomycetes Peronosporales**	**275 aa**	**5.60**	**1**	**114 bp**
*Saprolegnia diclina* (**XP_008621469**)	**Oomycetes Saprolegniales**	**268 aa**	**6.75**	**2**	**51 bp 47 bp**
*Aphanomyces invadans* (**XP_008869471**)	**Oomycetes Saprolegniales**	**270 aa**	**6.32**	**2**	**53 bp 88 bp**
*Albugo candida* (**CCI47927**)	**Oomycetes Albuginales**	**281 aa**	**7.62**	**3**	**49 bp 30 bp 57 bp**
*Guillardia theta* (**XP_005830762**)	**Cryptophyta Pyrenomonadales**	**200 aa**	**8.78**	**8**	**51 bp 158 bp 49 bp 47 bp 45 bp 50 bp 47 bp 51 bp**
*Nannochloropsis gaditana* (**EWM21023**)	**Eustigmatophyceae Eustigmatales**	**261 aa**	**9.78**	**2**	**160 bp 263 bp**
*Ectocarpus siliculosus* (**CBN78175**)	**Phaeophyceae Ectocarpales**	**141 aa**	**10.28**	**3**	**782 bp 1315 bp 1091 bp**
*Plasmodium fragile* (**XP_012337233**)	**Alveolata Aconoidasida**	**209 aa**	**9.48**	**0**^c^	**No introns**
*Toxoplasma gondii* (**KYF38827**)	**Alveolata Conoidasida**	**290 aa**	**9.39**	**0**	**No introns**
*Perkinsus marinus* (**XP_002786272**)	**Alveolata Perkinsida**	**188 aa**	**6.33**	**4**	**48 bp 59 bp 457 bp 200 bp**
*Bodo saltans* (**CUF18891**)	**Euglenozoa Kinetoplastida**	**163 aa**	**10.14**	**0**	**No introns**

a *Amborella trichopoda* is included as most basal Angiosperm representative.

b Phytozome accession number.

c Only introns in the protein coding region are counted.

**Table 2:** Features of BAP-like proteins and genes in the selected Uniconta species.

**Species name**	**Features of proteins and genes**				
**NCBI accession number**	**Taxon**	**Protein length**	**ProteinpI**	**Number of introns**	**Length of introns**
*Homo sapiens* BAP 31 **(EAW72818)**	**Chordata**	**246 aa**	**8.44**	**6**	**2181 bp 5183 bp 11448 bp 901 bp 828 bp 1032 bp**
	**Mammalia**				
*Homo sapiens* BAP 29 **(AAP35627)**	**Chordata**	**241 aa**	**9.55**	**6**	**3018 bp 9973 bp 1762 bp 4395 bp 2827 bp 8198 bp**
	**Mammalia**				
*Bos taurus* BAP31 **(AC_000187)**	**Chordata**	**245 aa**	**9.20**	**6**	**1421 bp 3620 bp 19548 bp 856 bp 795 bp 868 bp**
	**Mammalia**				
*Bos taurus* BAP29 **(NP_001033164)**	**Chordata Mammalia**	**240 aa**	**9.60**	**6**	**5274 bp 8892 bp 1426 bp 4620 bp 21723 bp 4683 bp**
*Egretta garzetta***(NW_009259250)**	**Chordata Aves**	**243 aa**	**9.11**	**6**	**3508 bp 2158 bp 4591 bp 2594 bp 7753 bp 1416 bp**
*Anolis carolinensis***(XP_003216939)**	**Chordata Lepidosauria**	**247 aa**	**8.74**	**6**	**1679 bp 14262 bp 4948 bp 10491 bp 6440 bp 8714 bp**
*Xenopus laevis* BAP29 **(AAH76818)**	**Chordata Amphibia**	**243 aa**	**9.39**	**6**	**2408 bp 1685 bp 2474 bp 795 bp 3921 bp 555 bp**
*Xenopus laevis* BAP31 **(NP_001086173)**	**Chordata Amphibia**	**244 aa**	**8.65**	**6**	**950 bp 4065 bp 3477 bp 273 bp 1233 bp 6242 bp**
*Danio rerio***(AAH49014)**	**Chordata Actinopterygii**	**247 aa**	**8.73**	**6**	**1202 bp 1868 bp 15175 bp 18469 bp 3950 bp 12955 bp**
	**Teleostei**				
*Latimeria chalumnae***(XP_005999374)**	**Chordata Actinopterygii**	**246 aa**	**8.90**	**4**	**10199 bp 16405 bp 1106 bp 20131 bp**
	**Coelacanthidae**				
*Ciona savignyi* (H2YK40^a^)	**Chordata Tunicata Ascidiacea**	**251 aa**	**6.84**	**6**	**1005 bp 789 bp 570 bp 469 bp 226 bp 1015 bp**
*Oikopleura dioica***(CBY14290)**	**Chordata Tunicata Appendicularia**	**235 aa**	**7.61**	**4**	**179 bp 47 bp 58 bp 47 bp**
*Branchiostoma floridae***(XP_002606682)**	**Chordata Cephalochordata Branchiostomidae**	**242 aa**	**6.98**	**6**	**512 bp 3061 bp 1594 bp 869 bp 450 bp 598 bp**
*Strongylocentrotus purpuratus***(XP_791898)**	**Echinodermata Eleutherozoa Echinozoa**	**161 aa**	**7.76**	**3**	**386 bp 826 bp 715 bp**
*Capitella teleta***(ELU03196)**	**Lophotrochozoa Annelida Polychaeta**	**236 aa**	**8.87**	**5**	**52 bp 105 bp 732 bp 3501 bp 54 bp**
*Helobdella robusta***(XP_009022177)**	**Lophotrochozoa Annelida Clitellata**	**234 aa**	**9.22**	**6**	**144 bp 249 bp 91 bp 3212 bp 116 bp 176 bp**
*Lingula anatine***(XP_013411827)**	**Lophotrochozoa Brachiopoda Linguliformea**	**244 aa**	**6.56**	**7**	**10030 bp 354 bp 2478 bp 123 bp 243 bp 209 bp 242 bp**
*Crassostrea gigas***(XP_011435954)**	**Lophotrochozoa Mollusca Bivalvia**	**237 aa**	**7.57**	**6**	**819 bp 2358 bp 677 bp 403 bp 613 bp 776 bp**
*Aplysia californica***(XP_005090966)**	**Lophotrochozoa Mollusca Gastropoda**	**246 aa**	**6.25**	**5**	**1043 bp 921 bp 5421 bp 332 bp 1603 bp**
*Lottia gigantea***(XP_009058881)**	**Lophotrochozoa Mollusca Gastropoda**	**237 aa**	**9.19**	**6**	**611 bp 1442 bp 446 bp 1115 bp 433 bp 591 bp**
*Biomphalaria glabrata***(XP_013084046)**	**Lophotrochozoa Mollusca Gastropoda**	**244 aa**	**6.41**	**5**	**988 bp 3021 bp 808 bp 146 bp 499 bp**
*Octopus bimaculoides***(XP_014771618)**	**Lophotrochozoa Mollusca Cephalopoda**	**240 aa**	**9.24**	**0**	**No introns**
*Philodina roseola***(ACI90387)**	**Lophotrochozoa Rotifera Bdelloidea**	**250 aa**	**9.44**	**6**	**73 bp 60 bp 50 bp 63 bp 95 bp 68 bp**
*Clonorchis sinensis***(GAA53952)**	**Platyhelminthes Trematoda**	**256 aa**	**7.80**	**7**	**345 bp 52 bp 4525 bp 415 bp 2538 bp 3249 bp 3650 bp**
	**Opisthorchiida**				
*Dictyocaulus viviparous***(KJH45191)**	**Ecdysozoa Nematoda Rhabditida**	**221 aa**	**8.71**	**5**	**171 bp 78 bp 68 bp 50 bp 186 bp**
*Caenorhabditis elegans***(NP_500267)**	**Ecdysozoa Nematoda Rhabditida**	**213 aa**	**9.55**	**2**	**109 bp 55 bp**
*Ascaris suum***(ERG85477)**	**Ecdysozoa Nematoda Ascaridida**	**235 aa**	**6.23**	**6**	**1187 bp 1089 bp 270 bp 626 bp 711 bp 315 bp**
*Wuchereria bancrofti***(EJW86459)**	**Ecdysozoa Nematoda Spirurida**	**210 aa**	**8.80**	**5**	**94 bp 121 bp 190 bp 209 bp 216 bp**
*Ixodes scapularis***(XP_002403043)**	**Ecdysozoa Arthropoda Acari**	**243 aa**	**9.08**	**7**	**106 bp 1304 bp 319 bp 248 bp 1219 bp 1795 bp 4918 bp**
*Parasteatoda tepidariorum***(XP_015910252)**	**Ecdysozoa Arthropoda Araneae**	**236 aa**	**8.69**	**6**	**2745 bp 2307 bp 2605 bp 2023 bp 2331 bp 7025 bp**
*Limulus polyphemus***(XP_013785233)**	**Ecdysozoa Arthropoda Merostomata**	**233 aa**	**8.39**	**6**	**5228 bp 3518 bp 2708 bp 673 bp 10338 bp 1550 bp**
*Daphnia pulex***(EFX80917)**	**Ecdysozoa Arthropoda Crustacea**	**236 aa**	**7.80**	**4**	**70 bp 95 bp 62 bp 62 bp**
*Hyalella azteca***(XP_018009243)**	**Ecdysozoa Arthropoda Crustacea**	**222 aa**	**8.84**	**3**	**502 bp 354 bp 224 bp**
*Orchesella cincta***(ODM98038)**	**Ecdysozoa Arthropoda Hexapoda Entomobryomorpha**	**226 aa**	**9.34**	**4**	**100 bp 78 bp 83 bp 83 bp**
*Bombyx mori***(XP_004923642)**	**Ecdysozoa Arthropoda Hexapoda Insecta**	**224 aa**	**8.51**	**0**	**No introns**
	**Lepidoptera**				
*Papilio machaon***(XP_014360347)**	**Ecdysozoa Arthropoda Hexapoda Insecta**	**222 aa**	**7.77**	**0**	**No introns**
	**Lepidoptera**				
*Plutella xylostella***(XP_011563833)**	**Ecdysozoa Arthropoda Hexapoda Insecta Lepidoptera**	**223 aa**	**8.81**	**0**	**No introns**
*Agrilus planipennis***(XP_018325264)**	**Ecdysozoa Arthropoda Hexapoda Insecta Coleoptera**	**228 aa**	**8.89**	**3**	**60 bp 63 bp 719 bp**
*Nicrophorus vespilloides***(XP_017778042)**	**Ecdysozoa Arthropoda Hexapoda Insecta Coleoptera**	**230 aa**	**9.22**	**2**	**101 bp 57 bp**
*Tribolium castaneum***(XP_015833546)**	**Ecdysozoa Arthropoda Hexapoda Insecta Coleoptera**	**238 aa**	**9.37**	**3**	**47 bp 1864 bp 50 bp**
*Drosophila elegans***(XP_017127683)**	**Ecdysozoa Arthropoda Hexapoda Insecta Diptera**	**228 aa**	**9.04**	**1**	**968 bp**
*Aedes albopictus***(KXJ82346)**	**Ecdysozoa Arthropoda Hexapoda Insecta Diptera**	**232 aa**	**9.46**	**2**	**9892 bp 208 bp**
*Musca domestica***(XP_005189051)**	**Ecdysozoa Arthropoda Hexapoda Insecta Diptera**	**233 aa**	**6.86**	**1**	**6715 bp**
*Limnephilus lunatus***(JDSM01012005)**	**Ecdysozoa Arthropoda Hexapoda Insecta Trichoptera**	**230 aa**	**9.02**	**0**	**No introns**
*Apis mellifera***(XP_397055)**	**Ecdysozoa Arthropoda Hexapoda Insecta Hymenoptera**	**229 aa**	**6.92**	**1**	**128 bp**
*Solenopsis invicta***(XP_011173333)**	**Ecdysozoa Arthropoda Hexapoda Insecta Hymenoptera**	**232 aa**	**9.46**	**1**	**78 bp**
*Polistes dominula***(XP_015172100)**	**Ecdysozoa Arthropoda Hexapoda Insecta Hymenoptera**	**234 aa**	**9.14**	**1**	**571 bp**
*Zootermopsis nevadensis***(**KK853387**)**	**Ecdysozoa Arthropoda Hexapoda Insecta Isoptera**	**231 aa**	**6.54**	**0**	**No introns**
*Acyrthosiphon pisum***(NP_001155477)**	**Ecdysozoa Arthropoda Hexapoda Insecta Hemiptera**	**212 aa**	**7.90**	**0**	**No introns**
*Pediculus humanus corporis***(XP_002429237)**	**Ecdysozoa Arthropoda Hexapoda Insecta Phthiraptera**	**224 aa**	**9.32**	**3**	**436 bp 92 bp 216 bp**
*Homalodisca liturata***(GECU01024317)**	**Ecdysozoa Arthropoda Hexapoda Insecta Hemiptera**	**229 aa**	**8.89**	**0**	**No introns**
*Exaiptasia pallida***(KXJ12234)**	**Cnidaria Anthozoa Hexacorallia Actiniaria**	**245 aa**	**6.39**	**5**	**167 bp 801 bp 134 bp 69 bp 454 bp**
*Acropora digitifera***(XP_015771945)**	**Cnidaria Anthozoa Hexacorallia Scleractinia**	**178 aa**	**4.93**	**5**	**114 bp 528 bp 3385 bp 456 bp 748 bp**
*Hydra vulgaris***(XP_004209484)**	**Cnidaria Hydrozoa**	**242 aa**	**9.32**	**4**	**5752 bp 18903 bp 9438 bp 75 bp**
*Intoshia linei***(OAF65985)**	**Mesozoa Orthonectida**	**220 aa**	**9.62**	**1**	**44 bp**
*Trichoplax adhaerens***(XP_002113615)**	**Placozoa Trichoplax**	**167 aa**	**9.34**	**3**	**1456 bp 340 bp 267 bp**
*Amphimedon queenslandica***(XP_011406596)**	**Porifera Demospongiae**	**172 aa**	**6.21**	**5**	**63 bp 1057 bp 47 bp 54 bp 107 bp**
*Monosiga brevicollis***(XP_001745234)**	**Choanoflagellida Codonosigidae**	**230 aa**	**9.04**	**4**	**189 bp 125 bp 139 bp 98 pb**
*Salpingoeca rosetta***(XP_004993388)**	**Choanoflagellida Salpingoecidae**	**185 aa**	**9.74**	**5**	**143 bp 251 bp 250 bp 239 bp 162 bp**
*Capsaspora owczarzaki***(XP_004345792)**	**Ichthyosporea Capsaspora**	**216 aa**	**9.43**	**6**	**151 bp 181 bp 87 bp 96 bp 171 bp 103 bp**
*Fonticula alba***(XP_009495214)**	**Nucleariidae and Fonticula group**	**226 aa**	**9.24**	**6**	**109 bp 176 bp 115 bp 148 bp 313 bp 232 bp**
*Saccharomyces cerevisiae* Yet3p **(AJU84316)**	**Fungi Dikarya Ascomycota Saccharomycotina**	**203 aa**	**5.93**	**0**	**No introns**
*Candida glabrata***(XP_445444)**	**Fungi Dikarya Ascomycota Saccharomycotina**	**191 aa**	**9.37**	**0**	**No introns**
*Penicillium italicum***(KGO75036)**	**Fungi Dikarya Ascomycota Pezizomycotina**	**210 aa**	**9.13**	**3**	**140 bp 57 bp 57 bp**
*Aspergillus calidoustus***(CEN62854)**	**Fungi Dikarya Ascomycota Pezizomycotina**	**213 aa**	**8.91**	**3**	**66 bp 51 bp 125 bp**
*Mycena chlorophos***(GAT59077)**	**Fungi Dikarya Basidiomycota Agaricomycotina**	**208 aa**	**9.36**	**6**	**55 bp 54 bp 54 bp 50 bp 57 bp 52 bp**
*Ustilago maydis***(XP_011388692)**	**Fungi Dikarya Basidiomycota Ustilaginomycotina**	**192 aa**	**9.42**	**2**	**82 bp 95 bp**
*Allomyces macrogynus***(KNE58291)**	**Fungi Blastocladiomycota Blastocladiomycetes**	**239 aa**	**6.99**	**4**	**81 bp 73 bp 80 bp 73 bp**
*Spizellomyces punctatus***(XP_016610704)**	**Fungi Chytridiomycota Chytridiomycetes**	**210 aa**	**9.33**	**9**	**72 bp 72 bp 61 bp 78 bp 46 bp 64 bp 64 bp 57 bp 62 bp**
*Gonapodya prolifera***(KXS12654)**	**Fungi Chytridiomycota Monoblepharido- mycetes**	**197 aa**	**7.89**	**6**	**66 bp 65 bp 76 bp 60 bp 64 bp 64 bp**
*Conidiobolus coronatus***(KXN68246)**	**Fungi Entomophthoro-mycota**	**207 aa**	**7.75**	**6**	**63 bp 42 bp 53 bp 54 bp 58 bp 52 bp**
*Rhizophagus irregularis***(ESA18893)**	**Fungi Glomeromycota Glomeromycetes**	**211 aa**	**7.77**	**8**	**85 bp 87 bp 72 bp 66 bp 68 bp 72 bp 81 bp 77 bp**
*Encephalitozoon intestinalis***(XP_003072841)**	**Fungi Microsporidia Unikaryonidae**	**199 aa**	**9.34**	**0**	**No introns**
*Edhazardia aedis***(EJW03664)**	**Fungi Microsporidia Edhazardia**	**192 aa**	**9.68**	**0**	**No introns**
*Nosema bombycis***(EOB11729)**	**Fungi Microsporidia Nosematidae**	**197 aa**	**9.30**	**0**	**No introns**
*Thecamonas trahens***(XP_013761453)**	**Apusozoa Apusomonadidae**	**150 aa**	**9.75**	**1**	**427 bp**
*Entamoeba invadens***(XP_004260006)**	**Amoebozoa Archamoebae Entamoebidae**	**167 aa**	**6.59**	**1**	**121 bp**
*Acanthamoeba castellanii***(XP_004337423)**	**Amoebozoa Discosea Longamoebia**	**223 aa**	**6.06**	**2**	**100 bp 280 bp**
*Polysphondylium pallidum***(EFA82930)**	**Amoebozoa Mycetozoa Dictyosteliida**	**206 aa**	**9.40**	**0**	**No introns**
*Acytostelium subglobosum***(XP_012752758)**	**Amoebozoa Mycetozoa Dictyosteliida**	**203 aa**	**9.40**	**0**	**No introns**
*Dictyostelium lacteum***(KYR02632)**	**Amoebozoa Mycetozoa Dictyosteliida**	**206 aa**	**9.37**	**0**	**No introns**

a UniProt (http://www.uniprot.org/) accession number.

## 2 Materials and methods

Annotation of the predicted genes and proteins was mined at the National Center for Biotechnology Information database (NCBI) and Phytozome database, version 11. Additional annotation of other predicted BAP-like proteins and genes was extracted from The UniProt Knowledgebase database (http://www.uniprot.org/).
