# Supplementary material for: Data on the exon-intron organization of genes coding for B-cell receptor-like proteins
Source: Data Brief. 2017 May 6;12:616–23. doi: 10.1016/j.dib.2017.05.011 (PMC5430146; doi:10.1016/j.dib.2017.05.011)
Supplement: Supplementary file 1 — Supplementary material [file mmc1.pdf]

Conflict of Interest Declaration

We wish to confirm that there are no known conflicts of interest associated with the publication "Exon-intron organization of the genes coding for B-cell receptor-like proteins" authored by Sergey Yu. Morozov, Anna V. Pankratenko, Anastasia K.Atabekova, and Andrey G. Solovyev.

We confirm that the manuscript has been read and approved by all named authors and that there are no other persons who satisfied the criteria for authorship but are not listed. We further confirm that the order of authors listed in the manuscript has been approved by all of us.

We understand that the Corresponding Author is the sole contact for the Editorial process (including Editorial Manager and direct communications with the office). He is responsible for communicating with the other authors about progress, submissions of revisions and final approval of proofs. We confirm that we have provided a current, correct email address which is accessible by the Corresponding Author.

Signed by all authors as follows:

Sergey Yu. Morozov	[signature]	30.03.2017
Anna V. Pankratenko	[signature]	31.03.2017
Anastasia K. Atabekova	[signature]	31.03.2017
Andrey G. Solovyev	[signature]	30.03.2017
